# Control of human cytomegalovirus replication by liver resident natural killer cells

**DOI:** 10.1038/s41467-023-37181-w

**Published:** 2023-03-14

**Authors:** Calum Forrest, Thomas J. G. Chase, Antonia O. Cuff, Dionas Maroulis, Reza Motallebzadeh, Amir Gander, Brian Davidson, Paul Griffiths, Victoria Male, Matthew Reeves

**Affiliations:** 1 Institute of Immunity & Transplantation, UCL, London, NW3 2PP UK; 2 Department of Surgery, UCL, Royal Free Campus, London, NW3 2PF UK; 3 Department of Metabolism, Digestion and Reproduction, Imperial College London, Chelsea & Westminster Campus, London, SW10 9NH UK

**Keywords:** Infection, Viral infection, Innate immune cells, Translational research, NK cells

## Abstract

Natural killer cells are considered to be important for control of human cytomegalovirus– a major pathogen in immune suppressed transplant patients. Viral infection promotes the development of an adaptive phenotype in circulating natural killer cells that changes their anti-viral function. In contrast, less is understood how natural killer cells that reside in tissue respond to viral infection. Here we show natural killer cells resident in the liver have an altered phenotype in cytomegalovirus infected individuals and display increased anti-viral activity against multiple viruses in vitro and identify and characterise a subset of natural killer cells responsible for control. Crucially, livers containing natural killer cells with better capacity to control cytomegalovirus replication in vitro are less likely to experience viraemia post-transplant. Taken together, these data suggest that virally induced expansion of tissue resident natural killer cells in the donor organ can reduce the chance of viraemia post-transplant.

## Abstract (web summary)

Natural killer cells play important roles in the immune response to human cytomegalovirus infection. Here the authors implicate donor-derived liver resident natural killer cells in the control of human cytomegalovirus infection by analysis of perfusates derived from human transplant livers.

## Introduction

Natural Killer (NK) cells have classically been considered innate immune cells, specialised in the recognition and killing of virally infected and malignant cells. There is now mounting evidence that NK cells, despite their innate classification, are capable of adaptive immune responses^1,2^. Individuals infected with human cytomegalovirus (HCMV) display an expanded subset of adaptive NK cells expressing increased NKG2C, CD2 and CD57, and decreased FcεRIγ^3–5^. Similarly, evidence of long-lived NK cell memory has been reported in murine CMV infections^6^. Despite emerging evidence from murine studies that liver-resident NK cells (lrNK) play a crucial role in the control of CMV infection, including the potential for adaptive responses to CMV^7,8^, most studies of NK cells have largely focused on their circulating counterparts.

NK cells represent only a small fraction of circulating lymphocytes, but account for up to 50% of lymphocytes in the human liver^9–11^. Approximately half of these NK cells are phenotypically and functionally similar to circulating NK cells and move freely between the liver and blood. The remaining NK cells express the tissue residency markers CD69 and CXCR6, are long-lived and unable to leave the liver^10–12^.These cells are largely found within the sinusoids^11^ and are thus easy to isolate from the perfusion fluid of livers destined for transplantation: they do not overlap with the smaller CD49a+ resident NK cell population that has been reported to be present in the parenchyma of some livers^13,14^. The preponderance of this large population of lrNK suggests important tissue-directed immunological functions, but these remain to be defined. Liver-resident NK cells are less able to kill malignant targets than their circulating counterparts^10^ but their ability to respond to virally infected cells remains uncharacterised.

Here we now show that in individuals naturally infected with HCMV there is an expansion of lrNK cells expressing an adaptive phenotype compared to that seen in individuals not infected with HCMV. Furthermore, we demonstrate that lrNK cells isolated from HCMV seropositive individuals are better able to control HCMV infection in vitro and that between donors differences in control were observed. Importantly, we observe that in patients who received livers with lrNK cells better able to control HCMV in vitro were less likely to experience post-transplant HCMV viraemia. Viral control and better outcomes post transplant correlate with a NKG2C + CD2 + sub-population of lrNK cells. Importantly, enrichment for CD2 + NK cells or blockade of CD2 activity could enhance or reduce control, respectively. Taken together, the data demonstrate that HCMV induces expansion of an adaptive phenotype in lrNK cells and that a CD2-expressing sub-population could play an important role for the regulation of HCMV in vivo.

## Results

### Tissue-resident NK cells in HCMV + individuals display an adaptive phenotype

**Fig. 1:**
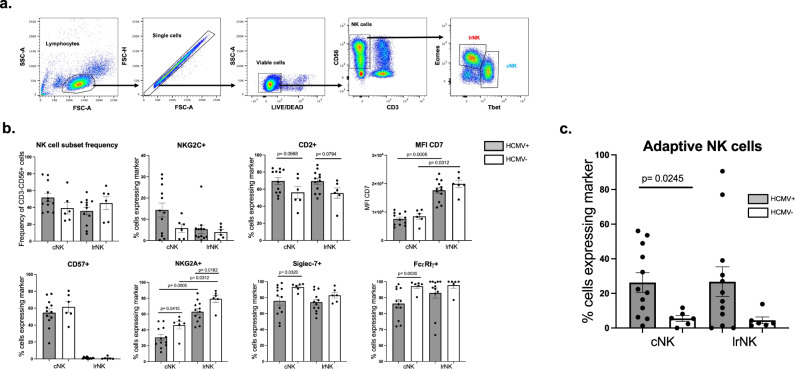
HCMV infection alters cNK and LrNK phenotypes. **a** NK cell subsets within liver perfusates (*n* = 18; 12 HCMV+, 6 HCMV-) could be separated into cNK and lrNK cells based on expression of the transcription factors Tbet and Eomes respectively. **b** cNK and lrNK cells were further examined for their expression of surface molecules associated with adaptive NK cells (CD57, NKG2C, NKG2A, CD2, CD7, Siglec7 and FcεRIγ). For all surface molecules except for CD7, frequencies of cells expressing the indicated molecule are shown. All cells were positive for CD7, so MFI is shown. **c** Co-staining for NKG2C, Siglec7 and FcεRIγ was used to examine abundance of adaptive NK cells (NKG2C + Siglec7- FcεRIγ -) within the cNK and lrNK population. Data are presented as mean values ± SEM. Significance was assessed using 2-tailed Wilcoxon’s signed-rank sum test (paired data) or a two-tailed Mann-Whitney U test (unpaired data) with *P* values less than 0.05 considered significant. Source Data are provided as a Source Data file.

Solid organ transplantation is a clinical setting in which HCMV poses a major risk. Indeed, the highest risk associated with HCMV is the introduction of HCMV with the donor organ (D+) into recipients with no prior infection of HCMV (R-). An intriguing observation from natural history studies is that despite increased risk not all R- individuals will develop viraemia upon transplant with a D+ organ^15^. Thus, we considered whether immunity transferred with the donor organ could contribute to outcome post-transplant. To begin to investigate the interaction of tissue-resident NK cells with viral infection we first examined both lrNK and cNK cells isolated from the perfusion fluid of livers destined for transplantation, allowing us to align phenotypic and functional analyses in vitro with HCMV serostatus and clinical outcomes in vivo. To do this, we first distinguished between cNK and lrNK cells on the basis of their expression of the transcription factors Tbet and Eomes, respectively^10^ (Fig. 1a) and then analysed expression of a number of adaptive NK cell markers. Consistent with a previous report^12^ we found that CD57 expression was restricted to cNK cells, while the lrNK cell population displayed an increased frequency of NKG2A-expressing cells and elevated CD7 and NKG2A expression (Fig. 1b; Supplementary Fig. 1). Although HCMV serostatus had no impact on the overall frequencies of cNK or lrNK cells (Fig. 1b), phenotypic changes including the expression of adaptive NK cell markers was observed in each of these populations, particularly in cNK cells (Fig. 1; Supplementary Fig. 1). HCMV seropositive individuals had increased frequencies of cNK expressing CD2 and decreased frequencies expressing the inhibitory receptors Siglec-7, NKG2A and the adaptor protein FcɛRIγ, consistent with previous observations for cNK in blood^5,16,17^. We observed similar trends for the frequency of expression of these markers in lrNK, with the decrease in frequency of NKG2A expression reaching significance (Fig. 1b). We also identified a subpopulation which co-expressed multiple markers consistent with an adaptive phenotype^5^ and found that this putative adaptive subpopulation was significantly enriched among cNK cells in HCMV seropositive donors, with a similar trend observed for lrNK cells (Fig. 1c). These observations were not restricted to NK cells within the liver but were also consistent within a similar analysis of NK cells from within kidney perfusion fluid (Supplementary Fig. 2). Furthermore, a phenotypic study of NK cells in the mucosa of the pregnant uterus (the decidua) also revealed changes in markers of adaptation (increased frequency of NKG2C+ (ns) and decreased frequencies of NKG2A+, Siglec7+ cells could be observed within HCMV+ individuals (Supplementary Fig. 3). Therefore, the role of HCMV in modulating immune cell populations is not restricted to peripheral blood but also to tissue resident cells within multiple and physiologically distinct tissues is important for HCMV pathogenesis.

### lrNK cells mediate better control of HCMV than cNK cells from the same donor

Although the phenotypic changes we observed were of interest it remained important to determine whether these resulted in functional differences in the ability of specific cells to control HCMV infection. Studies with MCMV have shown that expansion of adaptive NK cells provide protection from re-infection^6^. Furthermore, adaptive NK cells from HCMV seropositive donors are reported to be more likely to degranulate in response to HCMV-infected targets^5,18^ although these studies of human NK cells did not directly examine control of viral replication per se. Thus, our finding that among both cNK and tissue-resident NK cells, a subset of adaptive NK cells is expanded in HCMV seropositive individuals led us to investigate the anti-viral activity of both cNK and lrNK cells.

**Fig. 2:**
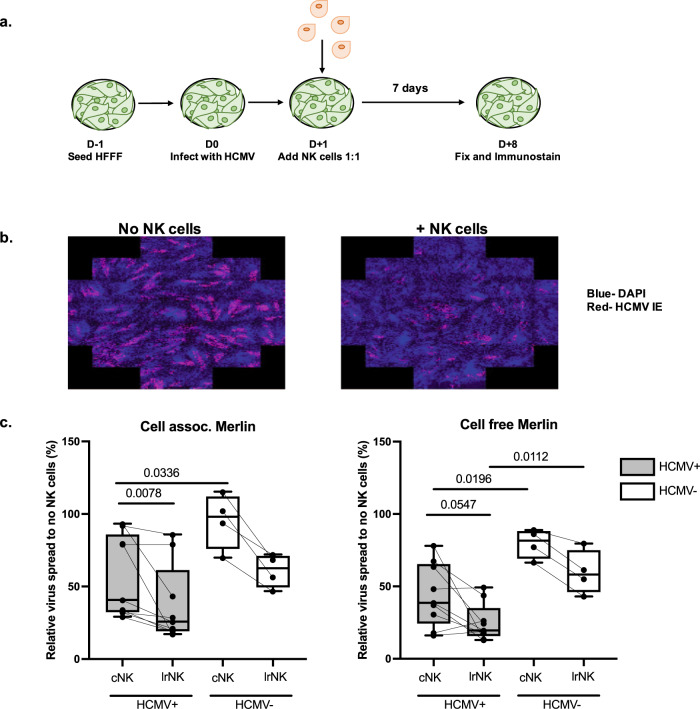
lrNK cells control HCMV replication in vitro. **a** Schematic of VDA. Seeded fibroblasts were infected with HCMV at ~0.01 MOI/well with sorted cNK or lrNK added after 24 h. After a further 7 days, cultures were fixed and immunostained for DAPI and HCMV-IE (secondary antibody on AF568). **b** Immunostained plates were analysed on a Hermes Wiscan scanning microscope with MetaMorph imaging software. Images were generated using tiles encompassing 50% of the well. **c** NK subset control of HCMV spread relative to infected cells without NK cells. Paired HCMV + (grey bars; individual donor *n* = 9) and HCMV- (open bars; individual donor *n* = 4) cNK and lrNK cell control of cell assoc. Merlin (left) and cell free Merlin (right). Data are shown as box and whisker plots showing line at median and min and max data points. Experiments were run once for each donor. Significance was assessed using two-tailed Wilcoxon’s signed-rank sum test (paired data) or a two-tailed Mann-Whitney *U* test (unpaired data) with *P* values less than 0.05 considered significant. Source Data are provided as a Source Data file.

In order to do this, we modified a viral dissemination assay (VDA) (Fig. 2a)^19^. Human foreskin fibroblasts (HFFs) were infected at a low multiplicity of infection (~0.01) with HCMV, and then NK cell subsets sorted at high purity^10^ (Supplementary Fig. 4) were introduced 24 h post infection. Seven days later, the co-cultures were fixed, the HFFs immunostained for HCMV-IE antigen (as a marker of viral spread in culture) and examined using high throughput microscopic analyses, allowing us to quantify the spread of infection (Fig. 2b). An important biological characteristic of HCMV is that it can transmit cell to cell through both cell-free (i.e. released from the producer cell into the supernatant) and cell-associated routes. In these experiments, we used two variants of the reference strain for wild type HCMV, Merlin. The ‘cell-free’ Merlin used in these studies has been passaged in vitro and thus grows predominantly cell-free, likely due to the acquisition of mutations that are not selected against in the absence of immune cells. The ‘cell associated’ Merlin is considered ‘wild type’ Merlin in that it has been re-engineered to more closely represent the wild type sequence^20^. Consequently, the growth of this virus is highly cell-associated and produces very little cell free virus.

Using these two variants of Merlin we observed that both cNK and lrNK cells isolated from HCMV + individuals (grey bars) displayed superior ability in their control of HCMV spread compared to their counterparts isolated from HCMV- individuals (clear bars) (Fig. 2c). Although variation between the ability of donor NK cells to control HCMV was observed, a paired analysis clearly showed that lrNK cells mediate better control of HCMV than cNK cells from the same donor (Fig. 2c). A similar trend was observed in HCMV- individuals but the limited availability of such donors likely prevented it reaching significance. Finally, it was evident that the ability of a donor’s NK cells to control HCMV was independent of the Merlin variant used (Supplementary Fig. 5). To test whether the response we saw was specific to HCMV infection we also set up a similar spread assay for herpes simplex-1 (HSV-1) infection (Supplementary Fig. 6). This assay revealed that lrNK cells appeared to control HSV-1 spread better than paired cNK cells, though due to the limited number of perfusates we were able to test, we were not able to test the significance. These results suggest that control by lrNK cells in our assay is not HCMV-specific but may be linked to lrNK cells having a generally enhanced antiviral function compared to cNK cells.

All our studies thus far had used NK cells that had not been pre-activated with cytokines, in contrast to previous studies on cNK^21,22^. Therefore, we considered whether lrNK may perform better simply because of prolonged exposure to the NK cell activating cytokine IL-15, which is highly expressed in the liver^23^. However, whilst IL-15 did enhance viral control, the enhancing effect was equally observed in both cNK and lrNK (Supplementary Fig. 7). This suggests that potential IL-15-mediated priming of lrNK in vivo cannot, by itself, account for the increased ability of lrNK to control HCMV and, further, suggests that the variation we observe between donors is unlikely to occur simply as a result of inter-donor variation in NK cell pre-activation. Consistent with IL-15 induced activation of both cNK and lrNK, elevated levels of IFNγ was detected post-stimulation—although differences in the capacity to produce the anti-viral cytokine IFNγ itself did not account for the inter-individual differences we observed despite being a marker for general activation of these cells (Supplementary Fig. 7).

### CD2 expression by lrNK correlates with their ability to control HCMV in vitro

A number of important observations were evident from the study so far: both liver-resident and circulating NK cell subsets are capable of controlling HCMV infection but the capacity to control in vitro varies between donors; furthermore, lrNK exert better control of HCMV than cNK, but control in both subsets is further enhanced in the HCMV + cohort. These differences led us to investigate whether the expression of specific adaptive markers on NK cells was associated with anti-viral activity in vitro, whether any such correlations differ between cNK and lrNK or depend on serostatus, with the ultimate aim of beginning to understand a mechanistic basis for anti-viral activity.

**Fig. 3:**
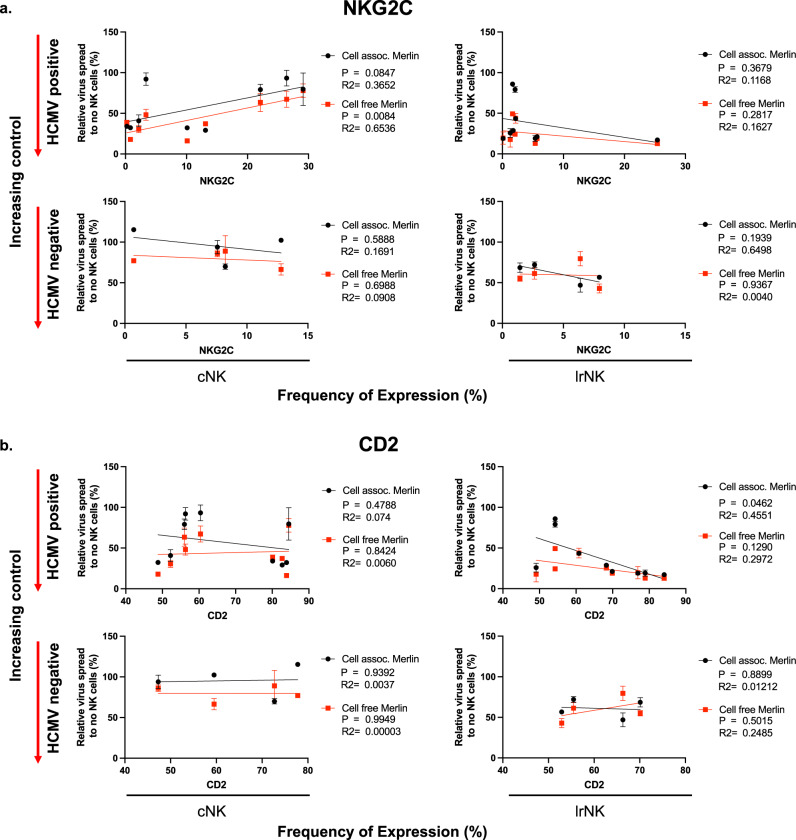
Control of cell-associated HCMV by lrNK cells directly correlates with frequency of CD2 + cells in seropositive individuals. In vitro control of HCMV strains (Cell assoc. merlin- Black, Cell free Merlin-Red) by cNK and lrNK from HCMV seropositive individuals (Top rows; *n* = 9) and seronegative (Bottom row; *n* = 4) was correlated against frequency of NK cells expressing NKG2C (**a**) and CD2 (**b**). Data are presented as mean values ± SEM. Significance was assessed using linear regression analysis with *P* values less than 0.05 considered significant. Source Data are provided as a Source Data file.

To identify potential subsets of NK cells responsible for control we performed a linear regression analysis of in vitro control on frequency of marker expression. This revealed two adaptive NK cell markers differentially associated with control: NKG2C and CD2 (Fig. 3 and Supplementary Table 1). The expansion of an NKG2C + population of cNK cells in HCMV infected individuals is well-established^4,5,24^ but, surprisingly, we found that the frequency of NKG2C + cNK cells was inversely related to the capacity of total cNK cells to control of HCMV in vitro (Fig. 3). This effect was only observed in the cNK cell population, with no correlation observed between in vitro control of virus and NKG2C expression on lrNK cells (Fig. 3) which may be expected given the very low frequency of NKG2C + lrNK cells (Fig. 1). CD2 is a co-stimulation/adhesion molecule that binds to CD58 (LFA3)—a reported target of the HCMV-encoded UL148 protein^25^. We observed that the frequency of CD2 + cells was positively correlated to in vitro protection for the lrNK cell population, with the strongest relationship seen in lrNK cells from seropositive donors (Fig. 3). In contrast, CD2 expression did not correlate with the capacity of cNK to control HCMV replication (Fig. 3 and Supplementary Fig. 8) suggesting that an accumulation of CD2 + tissue-resident NK cells may be an important component of the anti-viral activity we were observing.

### Frequency of CD2 + and NKG2C + lrNK cells correlates with HCMV control in transplant recipients

Although studies of immune function in vitro are highly instructive, it is desirable to determine whether this has any effect on protection in vivo. The strict species specificity of HCMV limits most analyses to in vitro studies, however, because the NK cells used in these experiments were isolated from the perfusion fluid of donor livers destined for transplantation, we were provided with a unique opportunity to investigate whether capacity to control HCMV in vitro was linked to post-transplant viral control in vivo. Crucially, liver transplant recipients at the Royal Free hospital are managed using pre-emptive valganciclovir therapy with patients only given anti-viral treatment when viraemia goes above a certain threshold^26^. This means we can assess the level of natural control of viral replication *prior* to treatment with anti-viral drugs. Thus, liver transplant recipients were monitored post-transplant for HCMV viraemia, which is a well-established surrogate marker of end organ disease and is the clinical measure that underpins the application of pre-emptive therapy to treat HCMV infection in vivo as well as HCMV clinical trials^26–29^.

**Fig. 4:**
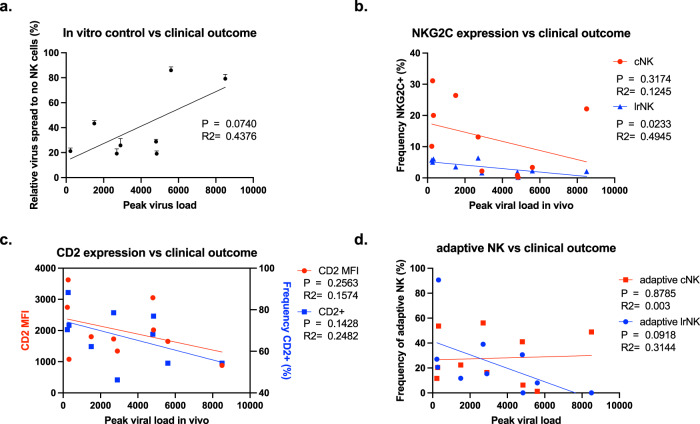
Increased frequency of ‘adaptive’ lrNK cells correlates with better outcome post-transplant. Peak viral load (genome copies/ml blood) within 1 year post-transplant in all transplant recipients who received a liver from a HCMV-seropositive donor is plotted against (**a**) in vitro control of HCMV spread by lrNK cells (black; *n* = 8). Expression of NKG2C + cNK (red) or lrNK (blue) cells (**b**), lrNK CD2 MFI (red)/frequency (blue) (**c**) or adaptive cNK (red) or lrNK (blue) cells (**d**) was plotted against peak viral load in vivo (*n* = 10). Significance was assessed using linear regression analysis with *P* values less than 0.05 considered significant. Source Data are provided as a Source Data file.

When patients were followed for up to one year post-transplant for development of HCMV viraemia, the capacity of lrNK cells to control HCMV in vitro was inversely correlated with peak viral load post-transplant when a liver from an HCMV + individual was transplanted (Fig. 4a). Most importantly, by examining the correlation between the expression of adaptive NK cell markers in the donor livers (Fig. 1) and clinical data on viral load in the recipients collected subsequently, we found that increased expression of CD2 and increased frequencies of CD2 + lrNK cells in the *donor* organ and increased frequencies of CD2 + cNK displayed a trend towards increased HCMV control in the *recipient* post-transplant (Fig. 4c; Supplementary Fig. 8b).

Interestingly we also observed that a higher frequency of NKG2C + lrNK cells was significantly associated with control in the recipient post-transplant (Fig. 4b), though this was not observed with cNK cells. Further, by examining the correlation between frequency of adaptive lrNK and cNK subsets (Fig. 1) versus clinical control we observed a trend for increased frequencies of adaptive lrNK but not adaptive cNK cells for increased HCMV control post transplant (Fig. 4d). Thus, lrNK with adaptive features of increased NKG2C and CD2 expression appear to display enhanced anti-viral function associated with clinical outcome in vivo.

### Control of HCMV by lrNK is mediated by CD2

Taken together, the in vitro data alongside the clinical data argued that CD2 + expression could have mechanistic importance but given the correlation with NKG2C + expression and clinical data, also implied NKG2C could play a role. Thus we interrogated both these molecules further.

**Fig. 5:**
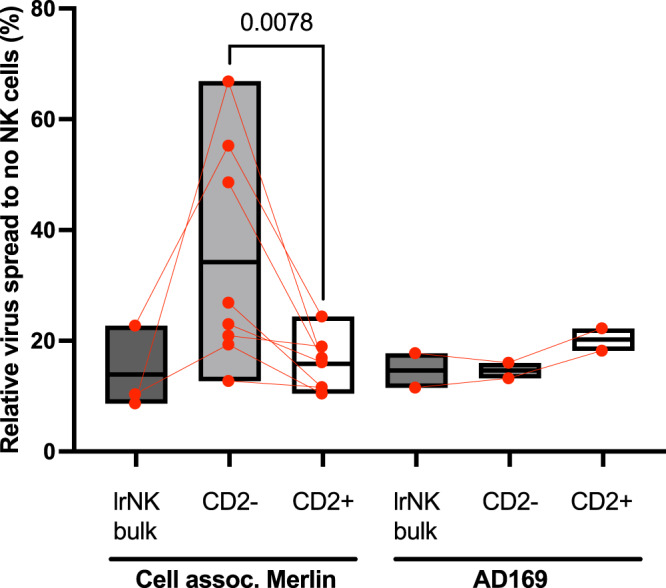
CD2 + lrNK cells control HCMV replication. Bulk lrNK cells or lrNK cells separated into CD2 + and CD2- fractions were analysed for their ability to control different HCMV strains in a virus dissemination assay (cell assoc. Merlin (individual donor *n* = 8) or AD169 (individual donor *n* = 2)). Data are presented as floating bars with individual values and showing mean values. Experiments were run once for each donor. Significance was assessed using a two-tailed Mann-Whitney *U* test with *P* values less than 0.05 considered significant. Source Data are provided as a Source Data file.

First, we flow sorted lrNK cells by their expression of CD2 and compared the CD2-positive and negative subpopulations against the donor’s paired bulk lrNK cells that still maintained heterogenous CD2 expression (Fig. 5). Control of HCMV spread was comparable between the bulk lrNK and the sorted CD2 + lrNK whereas this control was typically lower in the matched CD2- lrNK cell population. These data argued that the CD2 + population in the lrNK cells were an important component of anti-viral activity in our assays. In contrast to Merlin, the AD169 strain of HCMV (which has lost a number of NK immune evasion genes) was equally susceptible to control by CD2 + and CD2- lrNK cells, likely driven in part by its overall loss of immune evasion proteins targeting different NK cell pathways (Fig. 5).

**Fig. 6:**
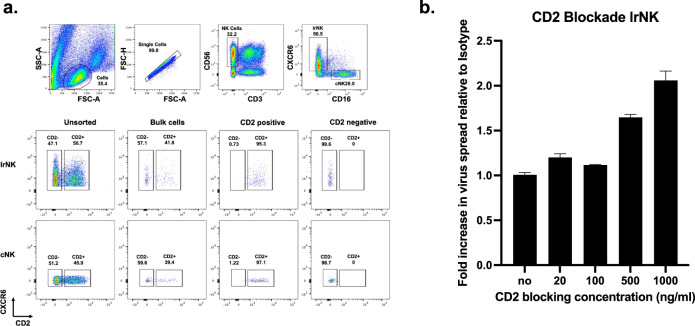
CD2 blockade inhibits control of HCMV infection by NK cells. **a** FACS sorted CD2 + lrNK or cNK cells were blocked (**b**) with aCD2 antibody or isotype control and then analysed for in vitro control of cell assoc. Merlin in a virus dissemination assay (replicates *n* = 2). Source Data are provided as a Source Data file.

Our data suggested that the CD2 + lrNK population was important for HCMV control so we tested whether this control was through CD2 directly. To investigate this, we once again sorted CD2 + lrNK as well as paired CD2 + cNK cells, blocked CD2 function using a monoclonal antibody and then examined their ability to control HCMV spread (Fig. 6 and Supplementary Fig. 9). At concentrations exceeding 500 ng/ml, blockade of CD2 reduced the level of control by both lrNK cells (Fig. 6) and paired cNK cells (Supplementary Fig. 9) against the cell associated Merlin. Therefore, control of cell associated HCMV spread by lrNK cells appears to be mediated at least partly through CD2. Notably, although blockade of CD2 on cNK cells did reduce their capacity to control HCMV spread, overall the control by cNK cells was still markedly reduced compared with paired lrNK.

In contrast, NKG2C blockade had no impact on in vitro control (Supplementary Fig. 10) suggesting a functional role for CD2 and a phenotypic correlation with NKG2C. The numbers of NKG2C + cells were low among lrNK (Fig. 1) meaning sorting experiments were only possible for cNK: among these, NKG2C + cells were equally effective at controlling HCMV as NKG2C- cells (Supplementary Fig. 11).

Interestingly, when we looked at associations between the expression of CD2 and that of other adaptive NK cell markers on lrNK cells, there was a positive association between CD2 (MFI and frequency of CD2 + lrNK) vs NKG2C MFI but not with frequency of NKG2C + lrNK cells (Supplementary Fig. 12). No associations were observed between CD2 and NKG2C expression on cNK cells (Supplementary Fig. 13). This could suggest an accessory or synergistic role of NKG2C with CD2 on lrNK cell activity but because only a small frequency of lrNK cells express NKG2C, formally testing this by examining the function of CD2 + NKG2C + versus CD2 + NKG2C- cells was not possible.

## Discussion

In this study we report that lrNK cells are anti-viral against a clinical isolate of HCMV in vitro, exerting greater anti-viral activity than cNK isolated from the same donor. Both lrNK and cNK cells from HCMV seropositive donors display increased expression of adaptive markers and increased ability to control the virus in vitro. This capacity for in vitro control by lrNK cells isolated from donor livers correlated with better control of HCMV post-transplant, as determined by peak viraemia, in the recipient. Critically, we were able to identify that expression of CD2, a costimulatory molecule on NK cells, was important in cellular control of HCMV spread and that increased expression of adaptive markers NKG2C and CD2 on lrNK cells was correlated with reduced viral loads post-transplant. Finally, we demonstrated that CD2 + NK cells limited HCMV spread in vitro and that blockade of CD2 reversed that control suggesting CD2 has a functional role in HCMV control by lrNK cells.

These findings have a number of important implications. Firstly, they support the idea that in humans, as in mice, the accumulation of adaptive NK cells in HCMV infected individuals can mediate protection against the virus and, furthermore, demonstrate the concept is valid in tissue-resident NK cells. Further, our observation that the ability of NK cells to control the virus was strongest among the lrNK cell population could reconcile previous findings in clinical situations where defective NK cells are associated with increased susceptibility to HCMV in vivo^30–32^, yet cNK cells are often reported to be poor at controlling viral replication in vitro^19,31^. We propose that a major NK cell type important for control of HCMV replication are, in fact, tissue-resident and have therefore not been considered in previous experiments on NK cell ability to control HCMV, which have used cells isolated from peripheral blood. The importance of tissue-resident NK cells would be consistent with the pathogenesis of HCMV, which replicates in tissues and then disseminates, and also congruent with the expression of multiple NK immune evasion genes by HCMV^33,34^. A number of NK immune evasion genes have been defined in studies using cNK cells but it is now clear that determining their role in regulating tissue-resident NK cells will reveal new insight into host-pathogen interactions – and resonates with the emerging concept that tissue-resident immunity is central for the control of CMV infection and, more broadly, other pathogens.^7,8,35–37^.

To test whether tissue-resident immunity was important against other pathogens we also repeated the virus dissemination assay using the alphaherpesvirus HSV-1. Interestingly we observed that lrNK cells also appeared to exert better antiviral function compared with paired cNK cells, though the limited number of perfusates available restricted the scope of this analysis. NK cells are also known to be important in control of alphaherpesviruses, with NK cell deficiencies resulting in life-threatening or fatal infections^38,39^, However, HSV-1 is greatly limited in the number of NK cell immune evasion proteins it expresses compared with HCMV^33^. This lack of virus specificity may highlight that CXCR6 + liver-resident NK cells has a broadly enhanced functional capacity. Recent work by the Paust lab have shown that ex vivo and expanded peripheral blood CXCR6 + NK cells phenotypically resemble CXCR6 + NK cells from the liver and that expanded CXCR6 + NK cells showed greater degranulation when stimulated with either K562 cells or PMA/ionomycin^40^.

Following on from our findings that lrNK expression of CD2 correlates with their ability to control HCMV in vitro and that the frequency of CD2 + and NKG2C + lrNK cells correlates with HCMV control in vivo, we sought to determine whether these molecules were required for HCMV control, or if they only act as markers of subsets responsible for control. Our findings that CD2- lrNK were less able to control HCMV than CD2 + lrNK, and that CD2 blockade reduces the ability of both lrNK and cNK to control HCMV spread pointed to CD2 as a key mediator of antiviral function. This is in line with studies of adaptive NK cells in the blood, which have shown CD2 to be elevated on in vitro expanded adaptive NK cells from HCMV + individuals^24^ and hypomethylation of the CD2 locus leading to increased transcription and protein expression^5^.

In contrast, we found no impact of NKG2C blockade on in vitro control and no difference in the ability of sorted NKG2C+ versus NKG2C- cNK to control HCMV in vitro. This could suggest that the correlation between NKG2C expression and viral control observed in vivo is a result of NKG2C identifying a subset that controls HCMV, although NKG2C itself is not required for this control. In support of this suggestion, individuals expressing a null variant of NKG2C are not at increased risk of HCMV^41^ and adaptive NK cells (identified by the expression of other adaptive markers) can be generated in these individuals^42^. In a study in stem cell transplant recipients, the frequency of adaptive NK cells post-reconstitution was associated with HCMV-free survival, but this was only significant when adaptive NK cells were defined by their expression of markers other than NKG2C^43^. Taken together with our findings that NKG2C is not necessary for viral control in vitro, this suggests that the observations by us and others^44^ that NKG2C + NK cells are protective against HCMV-free survival post-transplant may occur because NKG2C acts as a marker for adaptive NK cells, rather than because of any function of NKG2C itself.

CD2 is a major coactivating receptor expressed on T cells and NK cells that recognises CD58/ LFA-3 or weakly CD48 and can also synergise with CD16 and NKG2C to provide essential costimulatory signals and to lower the threshold for polyfunctional responses against UL40 peptide-loaded HLA-E^42,45^. This may partly explain why we see an association between expression of CD2 and NKG2C on lrNK cells. Other roles of CD2 include that as an adhesion molecule with expression linked to the formation of nanotubes between the NK cell and target cell that may serve to augment the ‘second signal’ provided by CD2 and thereby enhance NK cell cytotoxicity^46^. One possible scenario is that CD2 aids lrNK contact with their targets to allow enhanced antiviral control. Furthermore, CD2 may be less important in the cNK cell population because the mechanism underpinning the anti-viral activity of cNK cells may differ from lrNK cells. Thus, the attack of virally infected cells by NK cells is multi-faceted which, during multiple rounds of infection, may provide a selective environment to explain the accumulation of a comprehensive panel of NK evasion genes in HCMV including the presence of UL148 which has been reported to target LFA3/CD58 expression^33,34^.

Adaptive NK cells in the peripheral blood display enhanced ADCC, most likely through the loss of FcɛRIγ signalling and coupling to CD3ζ as well as costimulation through enhanced expression of CD2^16,42,47^. FcɛRIγ-NKG2C + adaptive NK cells have reduced levels of natural cytotoxicity receptors and weaker natural effector function against K562 cells whilst maintaining robust stimulation through CD16^16,47^. Therefore, the changes observed in NKG2C + adaptive blood NK cells appear to preferably direct their function toward responding to antibody-coated targets rather than through other NK cell activating receptors. An important aspect to note is that in this study we have analysed the functional activity of NK cells in the absence of any exogenous antibody that could mediate ADCC function. Therefore, it is reasonable to postulate that by seeding in relevant antibody we could see enhanced control of cNK cells and this might differ between HCMV + and HCMV- individuals. In contrast lrNK cells lack expression of CD16 and are therefore predicted to be unable to mediate ADCC, whilst maintaining the functional ability to respond through innate activating receptors. Therefore, the enhanced antiviral response to HCMV in seropositive individuals we observed here is independent of ADCC. Given that a number of our transplant recipients are HCMV seronegative there are likely to be few anti-HCMV antibodies available to mediate ADCC which may explain the stronger correlation with the lrNK cells and lack of correlation with NKG2C + cNK cells.

From a clinical perspective, the increased anti-viral activity of tissue-resident NK cells is likely to be particularly relevant in solid organ transplantation, in which the cohort of transplant recipients at greatest risk of HCMV viraemia are seronegative recipients with no HCMV immunity, receiving an organ from an HCMV seropositive donor. However, not all individuals in this cohort go on to develop HCMV disease, arguing that some level of control is exerted in those patients. With donor liver-resident NK cells shown to persist long-term up to 13 yrs post-transplant^10^, these donor cells could provide some ongoing protection in the recipient. The evidence presented here supports the hypothesis that tissue-resident immune cells in the incoming organ may be one source of viral control, with frequency of adaptive tissue-resident NK cells potentially an important biomarker for predicting which patients may require increased monitoring and/ or antiviral treatment. Whilst our focus has been on NK cells this does not rule out other tissue resident immune cells potentially playing a role and likely explains the stronger correlation between CD2 and in vitro control and the more complex picture in vivo – but definitely suggests tissue resident NK cells could be important. Another important caveat we have not addressed here is the biology of the target cell. Our studies are performed in fibroblasts but we cannot rule out HCMV is controlled by different ligands NK interactions in different cells and thus it will be interesting to expand analyses into a panel of primary cells.

This also becomes an important concept when considering strategies to control HCMV in multiple disease settings or to inform our understanding of developing NK cell immunotherapies. For example, if the concept of tissue-resident NK cell immunity extends into multiple tissues, we can develop strategies to induce this protective immune response through vaccination. This would be most beneficial in the context of congenital infection. Women infected during pregnancy can transmit HCMV to the foetus but this is less likely in women with HCMV immunity, which underpins the drive for a vaccine^48^. The uterine mucosa is a key site at which vertical transmission of the virus occurs^49^ and tissue-resident NK cells are particularly abundant here^50^. Here we report that NK cells with adaptive phenotypic features can be observed in the decidua of HCMV + individuals. Therefore, if HCMV protective tissue-resident NK cells can be identified and induced at this site through vaccination this would have significant public health implications. Clearly understanding the elements of tissue-resident immunity most important for viral control and, consequently, identifying strategies to induce them could become major considerations for future immune based therapies.

## Methods

### Cell isolation from perfusates

Perfusion fluid was obtained from 24 healthy livers (12 HCMV+; 6 HCMV-, 6 unknown serostatus) with post-transplant follow up with clinical outcome available for 17 recipients (11 HCMV+; 6 HCMV-). Leucocytes were isolated as previously^10^. Briefly, leucocytes were concentrated by centrifugation (750 × *g*, 15 min, 20 °C). The concentrated cells were layered onto Ficoll (GE Healthcare, Amersham, U.K.), centrifuged (400 × *g*, 20 min, 20 °C, light braking), and the interface was taken and washed twice with PBS (750 × *g*, 15 min, 20 °C). Isolated leucocytes were then aliquoted and cryopreserved in freezing media (90% FCS/ 10% DMSO) prior to use.

### Cell isolation from decidua

Decidua basalis was dissected in 2 × 2 cm sections with minimal placental tissue. Excess blood was washed away by stirring in cold PBS supplemented with 1% fetal calf serum for 20 min. Contaminating placental tissue was excised before centrifuging (300 × *g*, 10 min, RT). The pellet was homogenised in double weight per volume accutase enzymatic digestive medium on a GentleMACS machine, and shaking incubated at 37 °C (100 rpm, 1 h). The digest was passed through a 70 µm cell strainer before layering onto Histopaque and centrifuged (700 × *g*, 20 min, RT without brake). Leucocytes were collected within the interphase and twice washed in PBS with centrifugation (1st 700 × *g*, 10 min, RT and 2nd 500 × *g*, 10 min, RT).

### Serostatus analysis

Patient serostatus was confirmed by ELISA (Origene) completed on plasma isolated (1500 × *g*, 10 min, 4 °C) from matched blood collected in EDTA-coated blood tubes.

### Flow cytometry

Leucocytes from 18 liver perfusates (12 HCMV+; 6 HCMV-),9 kidney perfusates (4 HCMV+; 5 HCMV-) and 12 decidua (7 HCMV+; 5 HCMV-) were analysed for NK cell associated markers by flow cytometry. The following monoclonal antibodies were used: anti-human CD56 (clone NCAM16.2; 1:200 dilution) (supplied by BD Biosciences); anti-human CD3 (clone SK7; 1:200 dilution), CD16 (clone eBioCB16 (CB16); 1:200 dilution), Eomes (clone WD1928; 1:100 dilution), T-bet (clone eBio4B10 (4B10) 1:100 dilution) (all supplied by eBioscience); and anti-human CD159C (NKG2C) (clone Rea205; 1:100 dilution), CD159a (NKG2A) (clone REA110; 1:100 dilution) (all supplied by Miltenyi Biotec); and anti-human CD57 (clone HNK-1; 1:100 dilution), CD2 (RPA-2.10; 1:200 dilution), CD7 (clone CD7-6B7; 1:100 dilution), CD328 (Siglec-7) (clone 6-434; 1:200 dilution), CXCR6 (clone K041E5; 1:200 dilution) (all supplied by Biolegend); and anti-rat/human/mouse FcɛRIγ (polyclonal) (Supplied by Millipore; 1:100 dilution). Dead cells were excluded using fixable viability dye efluor 450 (eBioscience) or propidium iodide (PI) (Biolegend). For Intracellular staining, the Human FoxP3 Buffer (BD Biosciences) was used according to manufacturer’s instructions. Phenotypic analysis was carried out with data acquired on an LSRFortessa II (BD Biosciences) and analysed using FlowJo version 10.5.3 (FlowJo LLC, Becton Dickinson). FACS was carried out using a FACSAria (BD Biosciences) with all samples maintained at 4 °C throughout. liver-resident NK (LrNK) cells were isolated by sorting on live cells (PI), single cells, scatter and CD3^-^CD56^+^CD16^-^CXCR6^+^. Circulating NK (cNK) cells were isolated by sorting on live cells (PI), single cells, scatter and CD3^-^CD56^+^CD16^+^CXCR6^-^. Similarly, subsets of cNK or lrNK cells could be sorted into CD2+ and CD2- fractions.

#### CD2 and NKG2C blocking assays

A monoclonal mouse antibody against CD2 (clone RPA-2), NKG2C (MAB1381) or isotype controls (IgG1 or IgG2b, respectively) were used to block CD2 or NKG2C on flow-sorted NK cell populations A range of concentrations were used between 100–2000 ng/ml which is stated in the relevant figure. Cells were cultured for 30 min with titrated antibody in PBS. Cells were washed once and then resuspended in complete RPMI 1640 to a density of 5 × 10^5^ cells/ml.

### Virus dissemination assay (VDA)

Human Foreskin fibroblast (HFF; ATCC SRC-1041) cells were seeded to confluency in 96 well flat bottom plates in 100 µl/well culture media-DMEM (Gibco) supplemented with 10% FCS and Penicillin–Streptomycin (Thermo Fisher Scientific). The following day, HFFF cells were infected with viral strains (HCMV-Merlin Isolate, Merlin WT or AD169; or HSV-1) to achieve a level of infection of 1–5%^17^ in an additional 100 µl/well fresh culture media. After a following 24 h for HCMV or 6 h for HSV Sorted NK cell populations were added to the cultures. Briefly, frozen leucocytes from perfusion fluids were thawed and rested for 1 h prior to FACS in warm RPMI 1640 medium supplemented with 10% FCS and Penicillin-Streptomycin with the addition of DNAse I (0.1 mg/ml) (Sigma Aldrich). NK cell populations were isolated by FACS and resuspended in RPMI 1640 media to a density of 5 × 10^5^ cells/ml. Infected cell cultures were washed once with RPMI 1640 and 200 µl/ well NK cell fraction was added to infected HFFF cells. Infected cells without the addition of NK cells was used as a control. Cultures were allowed to run for a following 7 days for HCMV or 3 days for HSV-1.

### Immunostaining and microscopy

To end the VDA, cells were fixed and stained as previously^51^. Briefly, cells were fixed in 100% ice cold ethanol (20 min, −20 °C). Wells were washed once with DPBS and incubated (1 h, RT) with mouse anti-HCMV-IE (clone 6F8.2; Merck Millipore; 1:2000 dilution) or mouse anti-HSV-1-ICP4 (clone 10F1; Abcam; 1:2000 dilution) followed by incubation (1 h, RT) with DAPI and goat anti-mouse IgG AF568 (Thermo Fisher Scientific; 1:1000 dilution). Stained cells were quantified for viral IE protein expression by automated fluorescence microscopy and image recognition. A Hermes WiScan (IDEA Bio-Medical) automated microscope was used to capture 50% coverage/well with images processed using MetaMorph microscopy automation and image analysis software (Molecular Devices).

### Statistics and reproducibility

Relative infection in VDA were calculated from analysed microscopy images as N^o^ IE-positive DAPI-positive cells/Total N^o^ DAPI-positive cells. Graphs and statistical analyses were generated in GraphPad Prism version 8.4.0 (GraphPad Software). Summary data are shown as box and whisker plots (showing median and range). Significance was assessed using a two-tailed Wilcoxon’s signed-rank sum test or a two-tailed Mann-Whitney *U* test for comparisons of non-parametric data. Simple linear regression and Spearman rank was used to analyse statistical relationship or correlation in XY-plots. *P* values of less than or equal to 0.05 were taken to indicate a significant difference. No statistical method was used to predetermine sample size nor were any donor samples or data derived from them precluded from the analyses. The collection of tissue was randomised in that the only criterion was that perfusates were collected from the liver. The study was not blinded although the clinical data were obtained retrospectively once the initial in vitro studies of phenotype and anti-viral function had been performed.

#### Study approval

Ethical approval for use of perfusates, was obtained through the Royal Free Hospital Biobank (National Health Service Research Ethics Committee approval no. 11/WA/0077, study no. 9455). Perfusates are collected during the organ washing process that is performed in preparation for transplantation and thus are normally discarded. Decidua was dissected from the surface of placentae collected from women giving birth by caesarian section at term: these tissues would otherwise be discarded. The donors were informed that immune cells isolated from the surface of the placenta would be used to investigate immune control of viral infection during pregnancy and gave their written consent. The study was approved by the London—Chelsea Research Ethics Committee, study number 10/H0801/45. Donors received no remuneration for their tissues.

### Reporting summary

Further information on research design is available in the Nature Portfolio Reporting Summary linked to this article.

## Supplementary information

Supplementary Information

Reporting Summary

## Data Availability

The source data is provided in the supplementary data file. Any requests for additional data should be directed to the corresponding authors which will be made available. Source data are provided with this paper.

## Source data

Source Data
