# Supplementary material for: Control of human cytomegalovirus replication by liver resident natural killer cells
Source: Nat Commun. 2023 Mar 14;14:1409. doi: 10.1038/s41467-023-37181-w (PMC10014884; doi:10.1038/s41467-023-37181-w)
Supplement: Supplementary file 1 — Supplementary Information [file 41467_2023_37181_MOESM1_ESM.pdf]

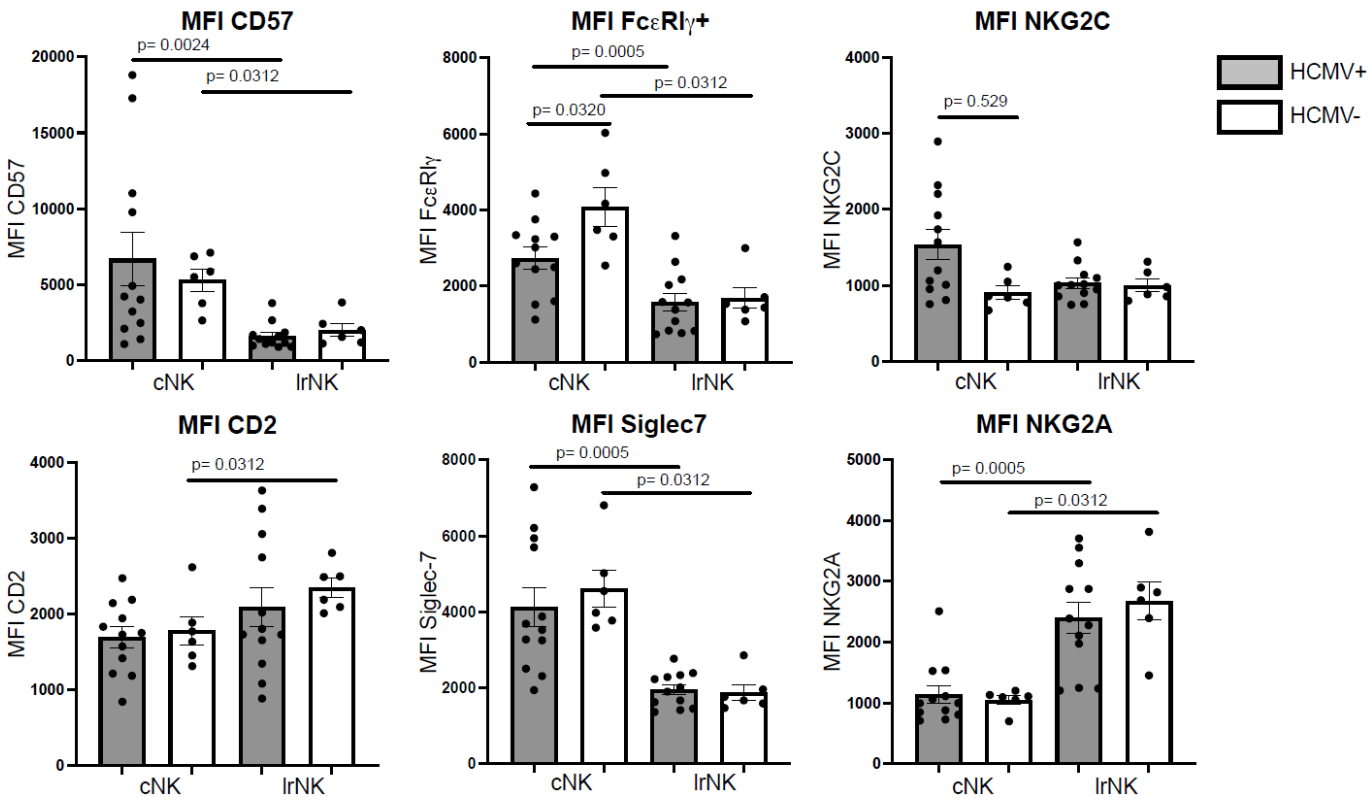

Figure S1. Expression phenotype of cNK or LrNK cells from HCMV seropositive or seronegative individuals. cNK and lrNK from HCMV seropositive (grey bars; n= 12) and seronegative (clear bars; n= 6) donors were analysed for their MFI expression of CD57, CD2, FcεRIγ, Siglec7, NKG2C, NKG2A. Significance was assessed using 2-tailed Wilcoxon's signed-rank sum test (paired data) or a 2-tailed Mann-Whitney U test (unpaired data) with P values less than 0.05 considered significant. Source Data are provided as a Source Data file.

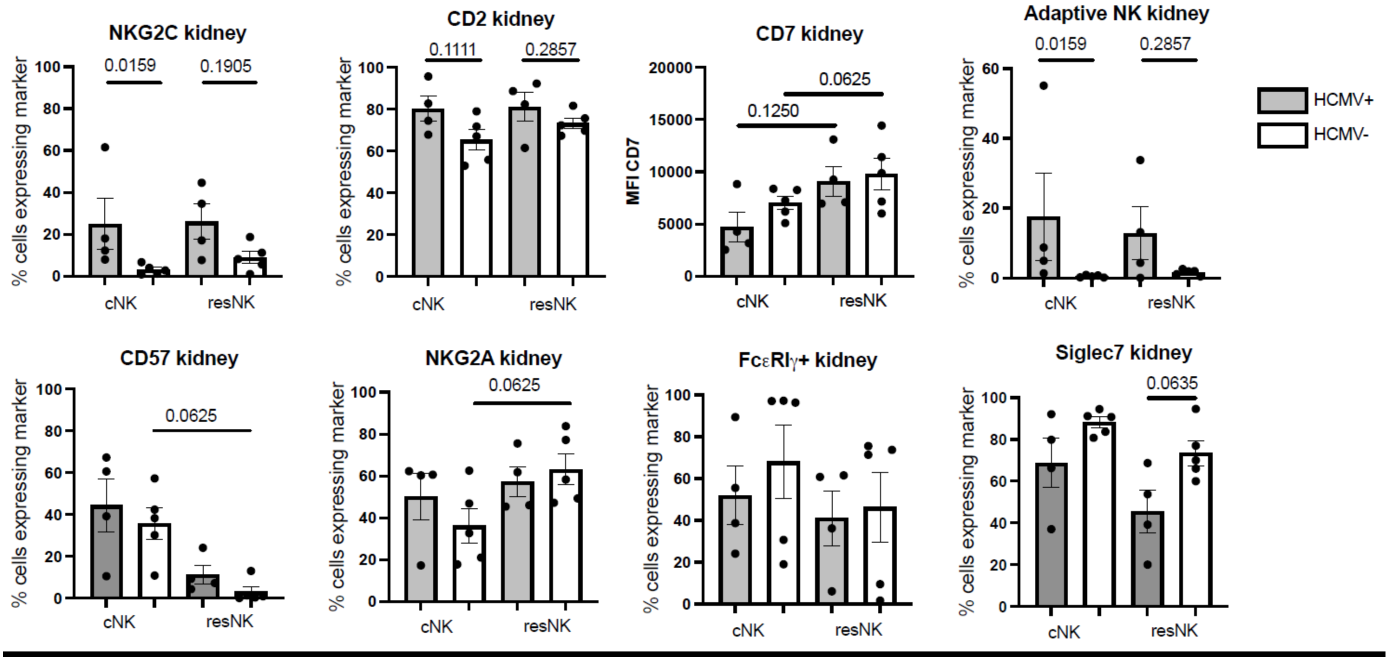

Figure S2. Expression phenotype observed in liver is recapitulated in the kidney. cNK and kidney-resident NK (krNK) from HCMV seropositive (grey bars; n= 4) and seronegative (clear bars; n= 5) donors were analysed for their MFI expression of CD57, CD2, FcεRIγ, Siglec7, NKG2C, NKG2A, and CD7. Significance was assessed using 2-tailed Wilcoxon's signed-rank sum test (paired data) or a 2-tailed Mann-Whitney U test (unpaired data) with P values less than 0.05 considered significant. Source Data are provided as a Source Data file.

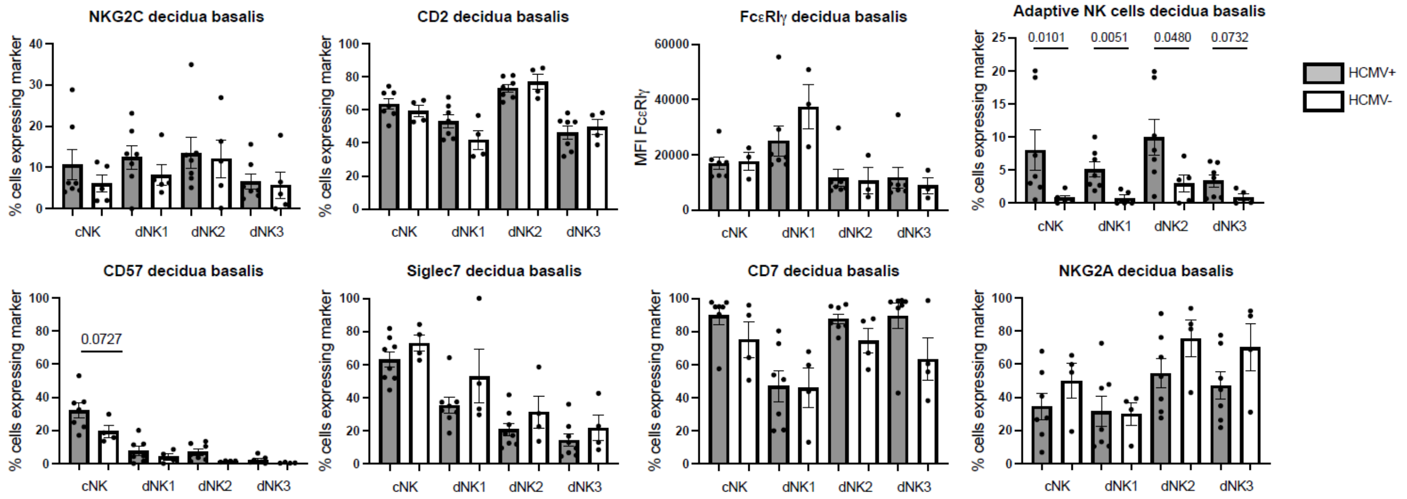

Figure S3. Expression phenotype of decidual NK cells from HCMV seropositive or seronegative individuals. Decidual NK cells from HCMV seropositive (grey bars; n= 7) and seronegative (clear bars; n= 5) were analysed for their MFI expression of CD57, CD2, FcεRIγ, Siglec7, NKG2C, NKG2A, and CD7. Significance was assessed using 2-tailed Wilcoxon's signed-rank sum test (paired data) or a 2-tailed Mann-Whitney U test (unpaired data) with P values less than 0.05 considered significant. Source Data are provided as a Source Data file.

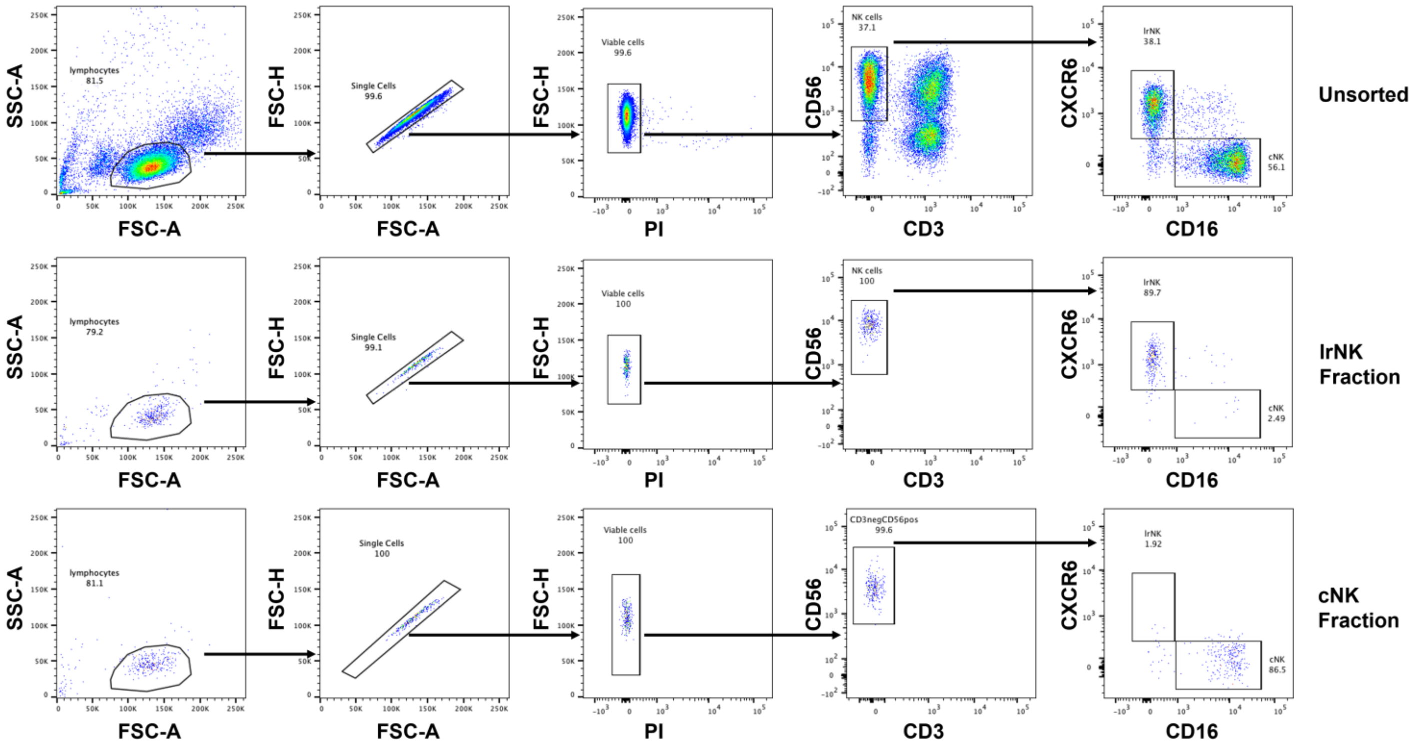

Figure S4. Gating strategy of sorted NK cell fractions from liver perfusates for use in functional assays. NK cells were separated from liver perfusates into cNK (CXCR6-CD16+) and LrNK (CXCR6+CD16) cell fractions by FACS. (Top) Unsorted lymphocyte fractions within perfusate. (Middle) Sorted LrNK cell fraction. (Bottom) Sorted cNK cell fraction.

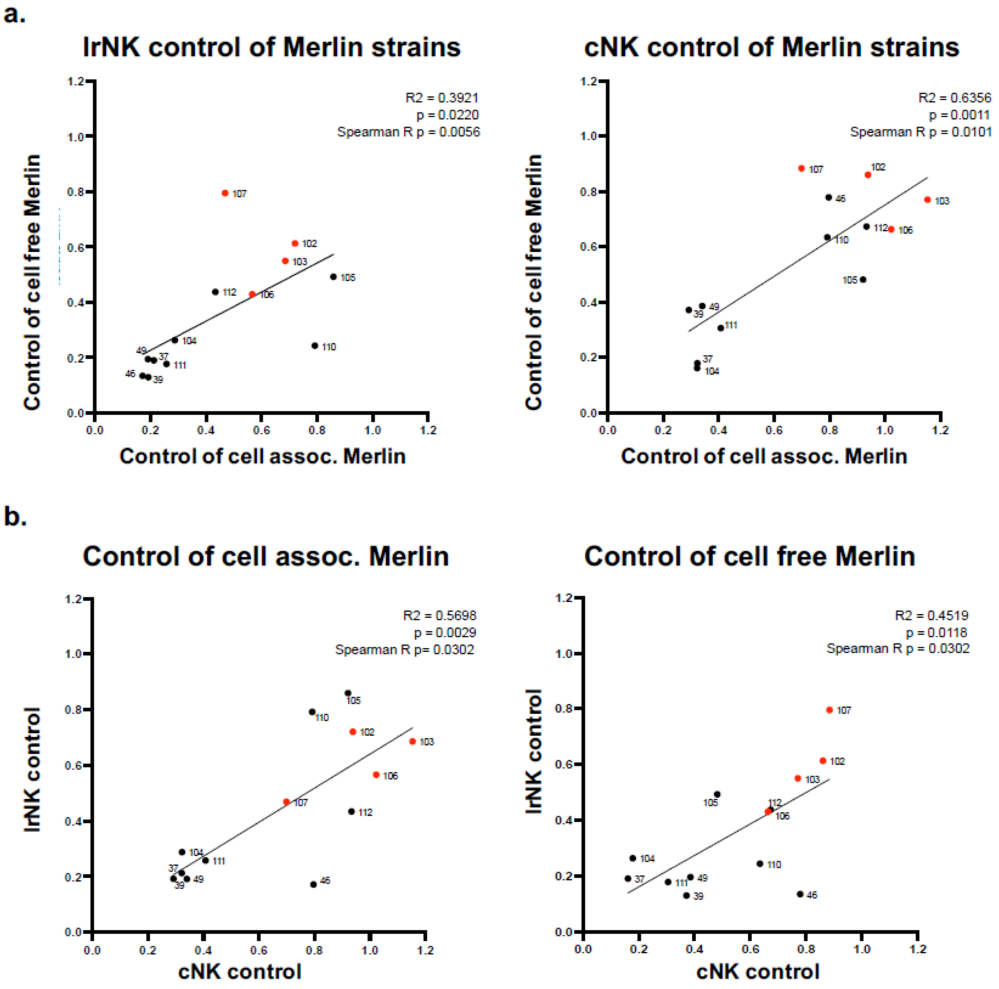

Figure S5. Correlation of NK cell mediated control between NK cell subsets and between HCMV strains. Data from virus spread assays plotted to assess correlation. HCMV seropositive donors (black; n= 9) and seronegative (red; n= 4) are plotted together. **(**a**)** Correlation between lrNK (left) and cNK (right) control of cell assoc. Merlin and cell free merlin. (b) Correlation between lrNK and cNK control of cell assoc. Merlin (left) and cell free merlin (right). Significance was assessed using linear regression analysis and Spearman's Rank correlation with P values less than 0.05 considered significant. Source Data are provided as a Source Data file.

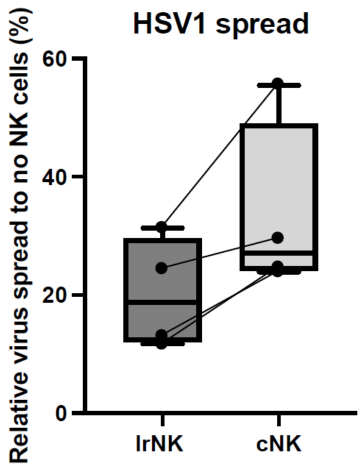

Fig S6. lrNK cells control HSV-1 replication in vitro. Seeded fibroblasts were infected with HSV-1 at ~0.01 MOI/well with sorted cNK or lrNK from donor perfusates (individual donors n=4) added after 6hrs. After a further 3days, cultures were fixed and immunostained for DAPI and HSV-1 ICP4 (secondary antibody on AF568). Immunostained plates were analysed on a Hermes Wiscan scanning microscope with MetaMorph imaging software. Data are shown as box and whisker plots showing line at median and min and max data points. Experiments were run once for each donor. Significance was not calculated due to limited sample size. Source Data are provided as a Source Data file.

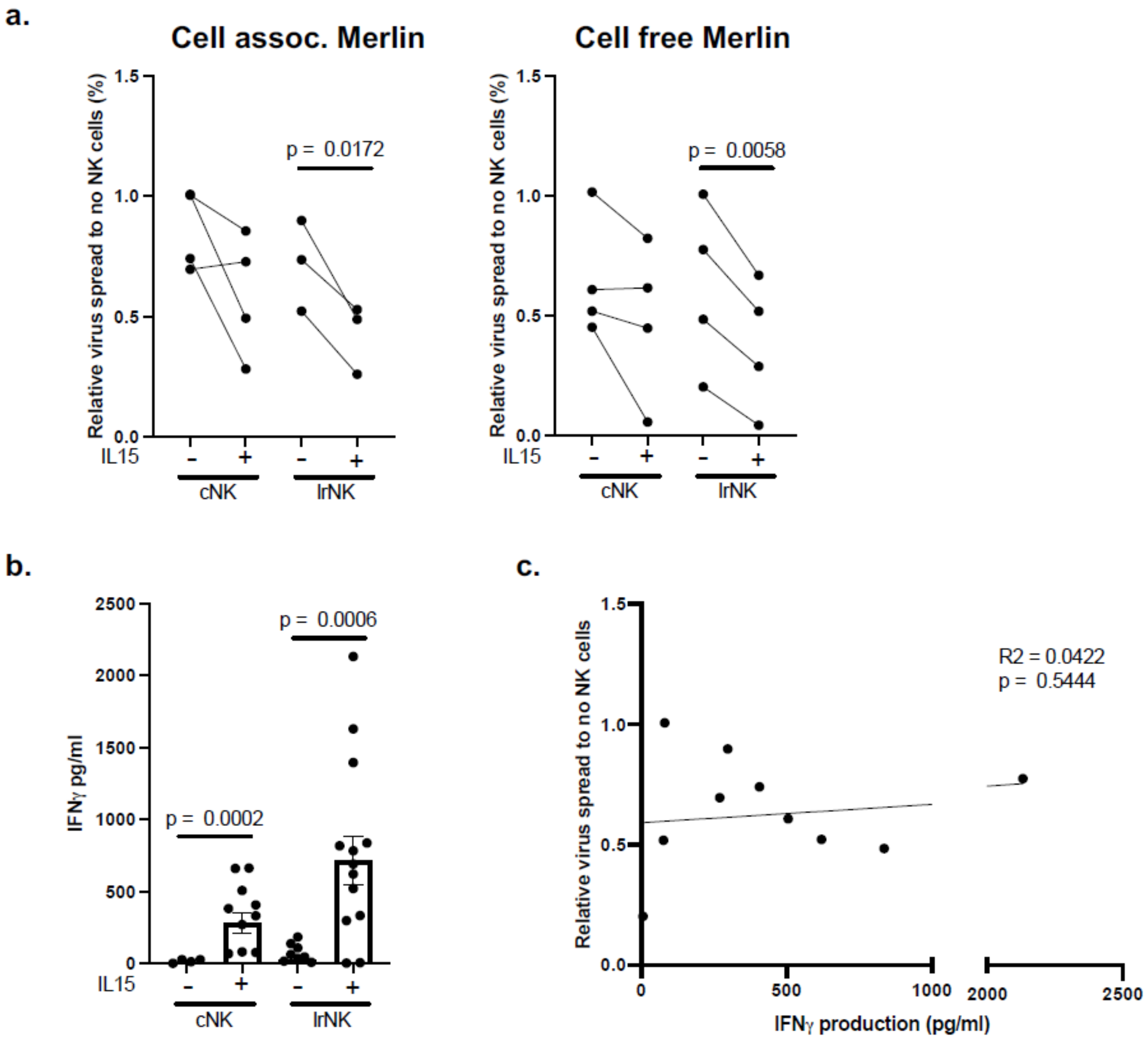

Fig S7. IL15 induced production of IFNγ is not associated with control. (a) cNK and lrNK were cultured with HCMV infected HFF alone or in the presence of 10ng/ml human recombinant IL15 (individual donors: cell assoc. cNK n= 4, lrNK n=3; cell free cNK n=4, lrNK n=4). (b) Culture supernatants were assessed for NK cell production of IFNγ by ELISA (paired replicates cNK vs cNK + IL15 n=12; lrNK vs lrNK + IL15 n=14) with data presented as mean values +/- SEM, and (c) levels of IFNγ were plotted against in vitro control (individual replicates n= 11). To determine the effects of IL-15 (a,b) we analysed the data using a repeated measures approach. Since repeated measures ANOVA cannot handle missing values, we fitted a mixed model, which uses a compound symmetry covariance matrix and is fitted using Restricted Maximum Likelihood (REML). Since measures were paired, rather than repeated, we assumed sphericity., (b) 2-tailed Wilcoxon's signed-rank sum test (paired data), or (c) linear regression analysis. Experiments were run once for each donor. P values less than 0.05 considered significant. Source Data are provided as a Source Data file.

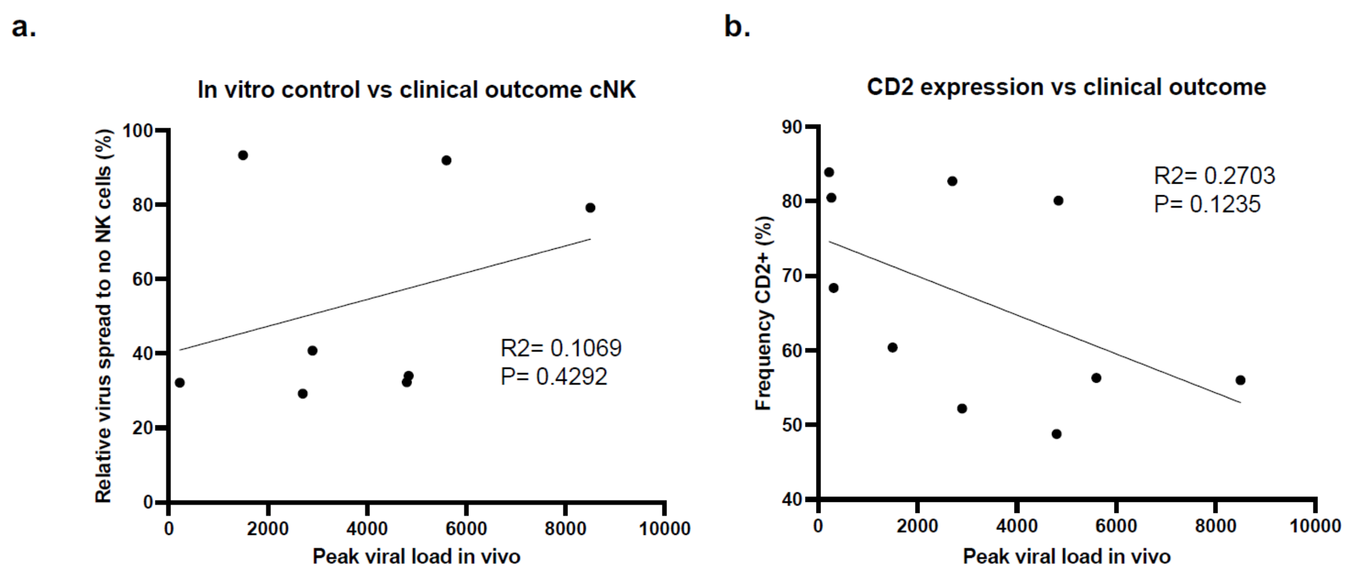

Figure S8. A trend was observed between cNK cells and post-transplant outcome. Peak viral load (genome copies/ml blood) within one year post-transplant in all transplant recipients who received a liver from a HCMV-seropositive donor is plotted against (a) *in vitro* control of HCMV spread by cNK cells (black; n= 8) or (b) frequency of CD2+ cNK cells (red: n=10) isolated from the donor liver prior to transplant. Significance was assessed using linear regression analysis. Source Data are provided as a Source Data file.

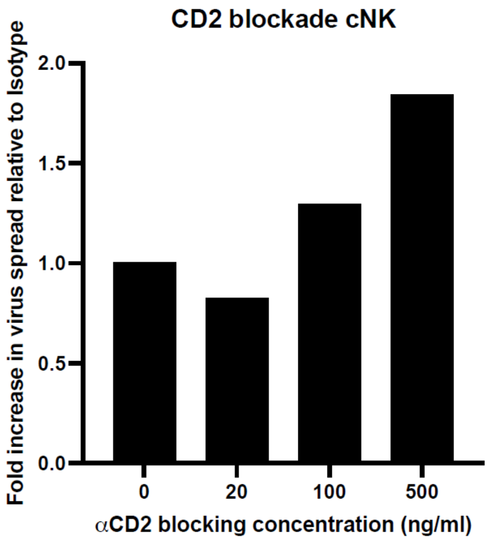

Figure S9. CD2 blockade inhibits control of HCMV infection by cNK cells. FACS sorted CD2+ cNK cells were blocked with aCD2 antibody or isotype control and then analysed for in vitro control of cell assoc. Merlin in a virus dissemination assay (n=1; in duplicate). Source Data are provided as a Source Data file.

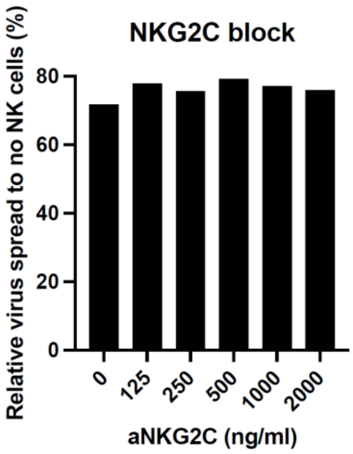

Figure S10. NKG2C blockade does not inibit control of HCMV infection by cNK cells. FACS sorted NKG2C+ cNK cells were blocked with aNKG2C antibody or isotype control and then analysed for in vitro control of cell assoc. Merlin in a virus dissemination assay (n=1; in duplicate). Source Data are provided as a Source Data file.

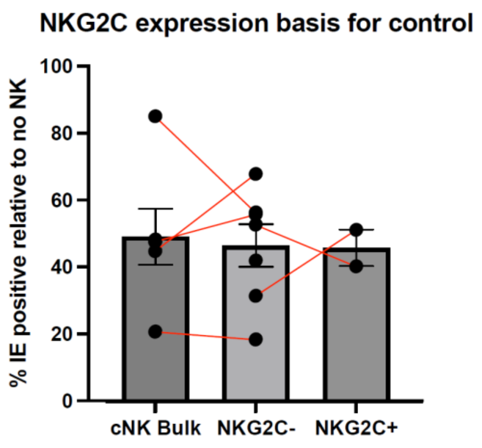

Figure S11. NKG2C+ and NKG2C- cNK cells control HCMV replication equally well. Bulk cNK cells (individual donor n=5) or cNK cells separated into NKG2C+ (individual donor n=7) and NKG2C- (individual donor n=2) fractions were analysed for their ability to control HCMV strain Merlin in a virus dissemination assay. Data are presented as mean values +/- SEM. Experiments were run once for each donor. Significance was assessed using a 2-tailed Mann-Whitney U test. Source Data are provided as a Source Data file.

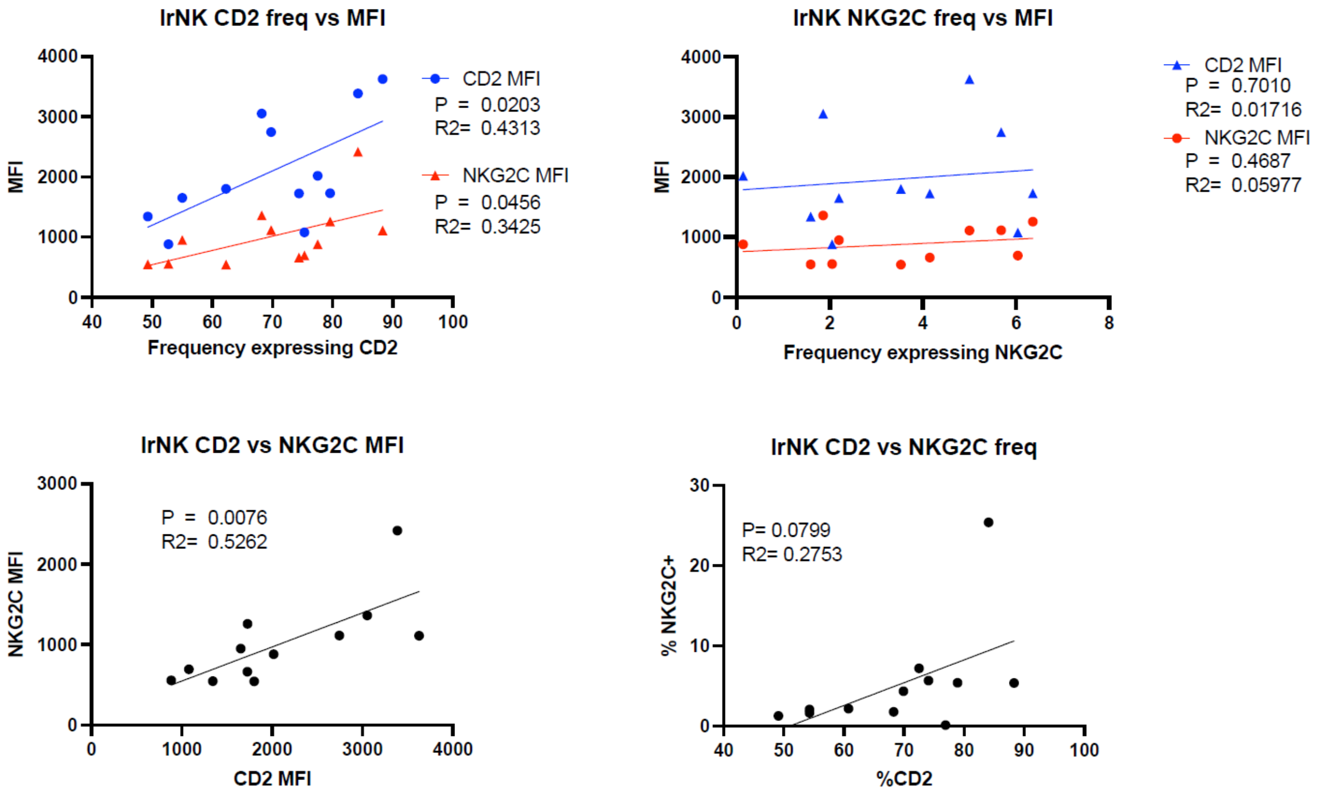

Figure S12. Association of NKG2C and CD2 expression by lrNK cells. Phenotypic analysis of NKG2C and CD2 expression on lrNK cells measured through frequency of positive cells or MFI. One donor perfusate was removed from top right graph due to outlying frequency of NKG2C+ lrNK cells (28.4% vs mean 3.51%). Significance was assessed using linear regression analysis. Source Data are provided as a Source Data file.

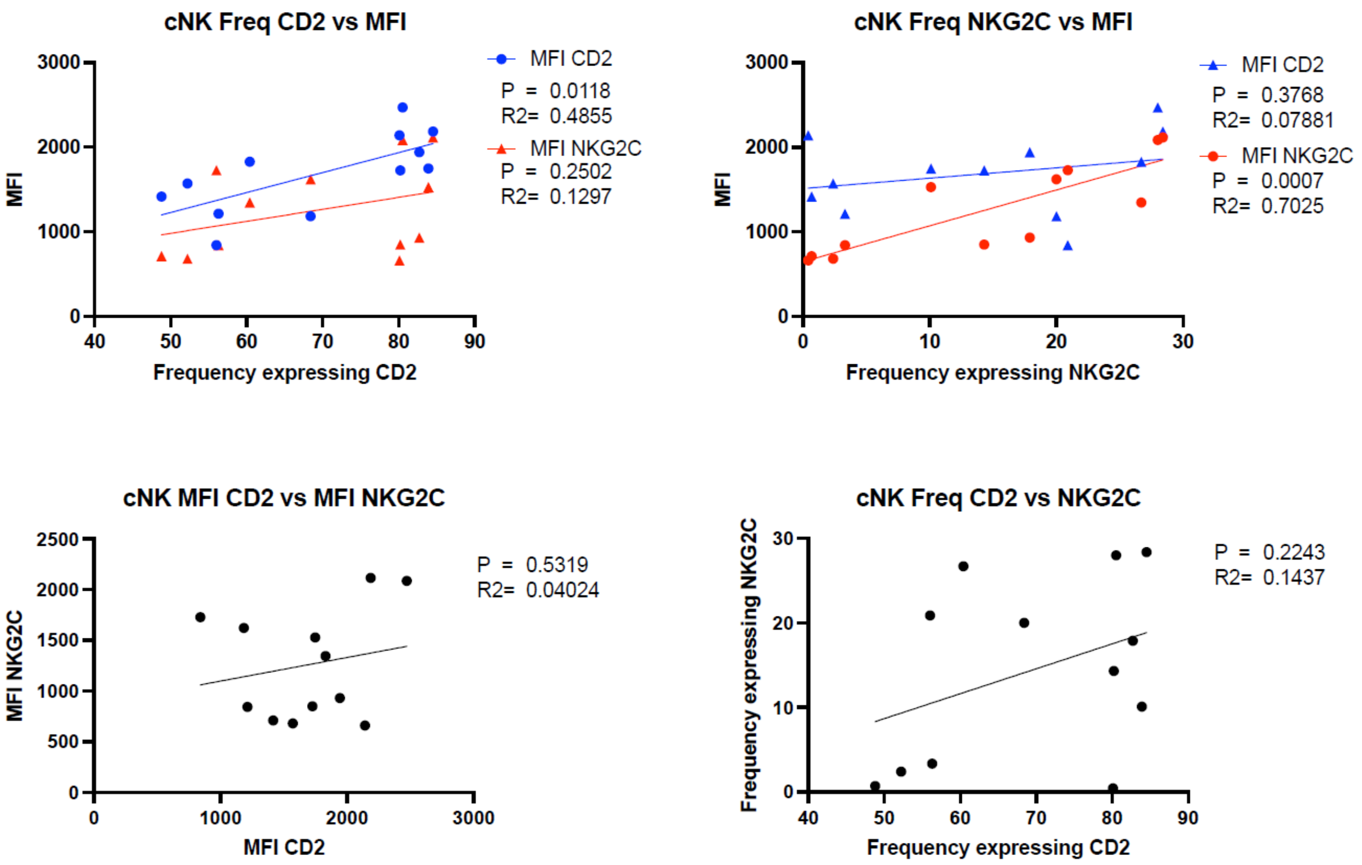

Figure S13. Association of NKG2C and CD2 expression by cNK cells. Phenotypic analysis of NKG2C and CD2 expression on cNK cells measured through frequency of positive cells or MFI. Significance was assessed using linear regression analysis. Source Data are provided as a Source Data file.

Freq (%)	Cell assoc. Merlin	HCMV+	HCMV-	
cNK	NKG2A	0.9282	0.1045	
	Siglec7	0.5281	0.0516	neg. assoc
	FcεRIγ	0.6141	0.1125	
	NKG2C	0.0847	0.5888	neg. assoc
	CD2	0.4788	0.9392	
	CD57	0.2087	0.7988	
lrNK	NKG2A	0.3788	0.0628	neg. assoc
	Siglec7	0.2105	0.5584	
	FcεRIγ	0.4355	0.4055	
	NKG2C	0.3679	0.1939	
	CD2	0.0462	0.1442	pos. assoc
	CD57	0.1308	0.8307	

Freq (%)	Cell free Merlin	HCMV+	HCMV-	
cNK	NKG2A	0.6759	0.2392	
	Siglec7	0.7321	0.1396	
	FcεRIγ	0.8909	0.4793	
	NKG2C	0.0084	0.6988	neg. assoc
	CD2	0.8424	0.9949	
	CD57	0.4219	0.889	
lrNK	NKG2A	0.1565	0.7905	
	Siglec7	0.0317	0.2933	neg. assoc
	FcεRIγ	0.7798	0.9228	
	NKG2C	0.2817	0.9367	
	CD2	0.129	0.9367	
	CD57	0.3588	0.2257	

MFI	Cell assoc. Merlin	HCMV+	HCMV-	
cNK	CD57	0.9654	0.6027	
	NKG2A	0.3018	0.4717	
	NKG2C	0.1052	0.7769	
	Siglec7	0.714	0.0396	neg. assoc
	CD7	0.788	0.2824	
	FcεRIγ	0.942	0.8407	
	CD2	0.4407	0.4891	
lrNK	CD57	0.9289	0.5167	
	NKG2A	0.1128	0.7774	
	NKG2C	0.7652	0.6159	
	Siglec7	0.5269	0.736	
	CD7	0.3087	0.2369	
	FcεRIγ	0.6289	0.2267	
	CD2	0.1027	0.7983	

MFI	Cell free Merlin	HCMV+	HCMV-	
cNK	CD57	0.6058	0.8971	
	NKG2A	0.8977	0.6851	
	NKG2C	0.1084	0.527	
	Siglec7	0.7303	0.2804	
	CD7	0.7431	0.0518	neg. assoc
	FcεRIγ	0.5193	0.3734	
	CD2	0.8794	0.7941	
lrNK	CD57	0.6912	0.9636	
	NKG2A	0.3005	0.813	
	NKG2C	0.8668	0.4154	
	Siglec7	0.0529	0.8924	neg. assoc
	CD7	0.4884	0.0936	neg. assoc
	FcεRIγ	0.3174	0.6344	
	CD2	0.4908	0.4433	

Linear Regression P value	<0.05	<0.1

Table S1. Association of NK cell phenotypic markers with in vitro control of HCMV. Expression frequency and MFI expression of NK cell markers CD57, CD2, FcεRIγ, Siglec7, NKG2C, NKG2A and CD7 were assessed for association with in vitro control of HCMV. Positive or negative association is indicated on relevant rows. Significance was assessed using linear regression analysis (p=<0.05 green, <0.1 yellow).
