# Supplementary material for: Control of human cytomegalovirus replication by liver resident natural killer cells
Source: Nat Commun. 2023 Mar 14;14:1409. doi: 10.1038/s41467-023-37181-w (PMC10014884; doi:10.1038/s41467-023-37181-w)
Supplement: Supplementary file 2 — Reporting Summary [file 41467_2023_37181_MOESM2_ESM.pdf]

nature portfolio

Corresponding author(s): Reeves; Male

Last updated by author(s): Feb 15, 2023

Reporting Summary

Nature Portfolio wishes to improve the reproducibility of the work that we publish. This form provides structure for consistency and transparency in reporting. For further information on Nature Portfolio policies, see our Editorial Policies and the Editorial Policy Checklist.

Statistics

For all statistical analyses, confirm that the following items are present in the figure legend, table legend, main text, or Methods section.

n/a	Confirmed	
☐	☒	The exact sample size (*n*) for each experimental group/condition, given as a discrete number and unit of measurement
☐	☒	A statement on whether measurements were taken from distinct samples or whether the same sample was measured repeatedly
☐	☒	The statistical test(s) used AND whether they are one- or two-sided<br>*Only common tests should be described solely by name; describe more complex techniques in the Methods section.*
☒	☐	A description of all covariates tested
☐	☒	A description of any assumptions or corrections, such as tests of normality and adjustment for multiple comparisons
☐	☒	A full description of the statistical parameters including central tendency (e.g. means) or other basic estimates (e.g. regression coefficient) AND variation (e.g. standard deviation) or associated estimates of uncertainty (e.g. confidence intervals)
☐	☒	For null hypothesis testing, the test statistic (e.g. F, t, r) with confidence intervals, effect sizes, degrees of freedom and P value noted<br>*Give P values as exact values whenever suitable.*
☒	☐	For Bayesian analysis, information on the choice of priors and Markov chain Monte Carlo settings
☒	☐	For hierarchical and complex designs, identification of the appropriate level for tests and full reporting of outcomes
☒	☐	Estimates of effect sizes (e.g. Cohen's *d*, Pearson's *r*), indicating how they were calculated

Our web collection on statistics for biologists contains articles on many of the points above.

Software and code

Policy information about availability of computer code

Data collection	Percentage infection was assessed by automated fluorescence microscopy and image recognition Hermes WiScan (IDEA Bio-medical) instruments and processed by Metamorph software (Molecular Devices)<br>Phenotypic analysis was carried out with data acquired on an LSRFortessa II, FACSAria-llu or FACSAria-fusion (BD Biosciences)
Data analysis	Data analyses was performed using Graphpad Prism software (version 8.4.0). Flow cytometry data was analysed using FlowJo version 10.7.2

For manuscripts utilizing custom algorithms or software that are central to the research but not yet described in published literature, software must be made available to editors and reviewers. We strongly encourage code deposition in a community repository (e.g. GitHub). See the Nature Portfolio guidelines for submitting code & software for further information.

Data

Policy information about availability of data

All manuscripts must include a data availability statement. This statement should provide the following information, where applicable:

- Accession codes, unique identifiers, or web links for publicly available datasets
- A description of any restrictions on data availability
- For clinical datasets or third party data, please ensure that the statement adheres to our policy

The underlying data of the figures shown in the manuscript is provided in the source file. We have also added that any additional requests for data can be directed to the authors

Human research participants

Policy information about studies involving human research participants and Sex and Gender in Research.

Reporting on sex and gender	We have not performed studies on human participants but discarded tissue from those individuals. We collected no data on the sex or gender of the liver and renal perfusate samples. The samples were collected anonymised with no identifiable data. The Decidua cells were collected from females only (collected from placenta removed after caesarian section)
Population characteristics	These samples are either from organ transplant and we did not collect data on the population beyond CMV status or from females for the decidua study (see above)
Recruitment	for transplant perfusates these are the waste material collected during preparation of the organ for transplant. Livers and some kidneys are from deceased patients. Where possible (live renal and decidua) informed consent was given.
Ethics oversight	Ethical approval for use of perfusates, was obtained through the Royal Free Hospital Biobank (National Health Service Research Ethics Committee approval no. 11/WA/0077, study no. 9455). Perfusates are collected during the organ washing process that is performed in preparation for transplantation and thus are normally discarded. Decidua was dissected from the surface of placentae collected from women giving birth by caesarian section at term: these tissues would otherwise be discarded. The donors were informed that immune cells isolated from the surface of the placenta would be used to investigate immune control of viral infection during pregnancy and gave their written consent. The study was approved by the London - Chelsea Research Ethics Committee, study number 10/H0801/45. Donors received no remuneration for their tissues.

Note that full information on the approval of the study protocol must also be provided in the manuscript.

Field-specific reporting

Please select the one below that is the best fit for your research. If you are not sure, read the appropriate sections before making your selection.

☒ Life sciences ☐ Behavioural & social sciences ☐ Ecological, evolutionary & environmental sciences

For a reference copy of the document with all sections, see nature.com/documents/nr-reporting-summary-flat.pdf

Life sciences study design

All studies must disclose on these points even when the disclosure is negative.

Sample size	sample sizes were determined by the number of perfusates we could collect and were available for study
Data exclusions	no data exclusions were made. We did not have enough cells from all perfusates to perform all experiments but no data collected was excluded from any analyses
Replication	multiple donors were analysed in duplicate/triplicate and over multiple experiments (cell number permitting)
Randomization	The only segregation performed for analyses was based on donor organ. HCMV serostatus and viraemia was allocated after analyses
Blinding	as stated above we analysed all samples when they became available and then retrospectively identified HCMV serostatus and any clinical information if the donor organ proceeded for use in transplant.

Reporting for specific materials, systems and methods

We require information from authors about some types of materials, experimental systems and methods used in many studies. Here, indicate whether each material, system or method listed is relevant to your study. If you are not sure if a list item applies to your research, read the appropriate section before selecting a response.

Materials & experimental systems

n/a	Involved in the study
	[x] Antibodies
	[x] Eukaryotic cell lines
[x]	Palaeontology and archaeology
[x]	Animals and other organisms
	[x] Clinical data
[x]	Dual use research of concern

Methods

n/a	Involved in the study
[x]	ChIP-seq
	[x] Flow cytometry
[x]	MRI-based neuroimaging

Antibodies

Antibodies used	anti-human CD56 (clone NCAM16.2; 1:200 dilution) (supplied by BD Biosciences); anti-human CD3 (clone SK7; 1:200 dilution), CD16 (clone eBioCB16 (CB16); 1:200 dilution), Eomes (clone WD1928; 1:100 dilution), T-bet (clone eBio4B10 (4B10) 1:100 dilution) (all supplied by eBioscience); and anti-human CD159C (NKG2C) (clone Rea205; 1:100 dilution), CD159a (NKG2A) (clone REA110; 1:100 dilution) (all supplied by Miltenyi Biotec); and anti-human CD57 (clone HNK-1; 1:100 dilution), CD2 (RPA-2.10; 1:200 dilution), CD7 (clone CD7-6B7; 1:100 dilution), CD328 (Siglec-7) (clone 6-434; 1:200 dilution), CXCR6 (clone K041E5; 1:200 dilution) (all supplied by Biolegend); and anti-rat/human/mouse FcεRIγ (polyclonal) (Supplied by Millipore; 1:100 dilution). Dead cells were excluded using fixable viability dye efluor 450 (eBioscience) or propidium iodide (PI) (Biolegend). Mouse anti-NKG2C (MAB1381; biotechne 100-2ug/ml).<br>anti-HCMV-IE (clone 8B16F8.2; Merck Millipore; 1:2000 dilution)<br>mouse anti-HSV-1-ICP4 (clone 10F1; Abcam; 1:2000 dilution)<br>goat anti-mouse IgG AF568 (Thermo Fisher Scientific; 1:1000 dilution)
Validation	Each commercial antibody is used extensively in our lab and was, and is, used based on website verification and our own in house analyses

Eukaryotic cell lines

Policy information about cell lines and Sex and Gender in Research

Cell line source(s)	ATCC - HFFs (SRC-1041)
Authentication	The cell line was established in 2003 by ATCC details on validation of their cell lines is found at: https://www.atcc.org/cell-products/human-cells#t=productTab&numberOfResults=24
Mycoplasma contamination	We routinely check for mycoplasma in the lab and the cells were confirmed mycoplasma free at time of analysis
Commonly misidentified lines (See ICLAC register)	no commonly misidentified cell lines were used

Clinical data

Policy information about clinical studies

All manuscripts should comply with the ICMJEguidelines for publication of clinical research and a completedCONSORT checklist must be included with all submissions.

Clinical trial registration	this is not a clinical trial
Study protocol	n/a
Data collection	n/a
Outcomes	n/a

Flow Cytometry

Plots

Confirm that:

- [x] The axis labels state the marker and fluorochrome used (e.g. CD4-FITC).
- [x] The axis scales are clearly visible. Include numbers along axes only for bottom left plot of group (a 'group' is an analysis of identical markers).
- [x] All plots are contour plots with outliers or pseudocolor plots.
- [x] A numerical value for number of cells or percentage (with statistics) is provided.

Methodology

Sample preparation	Cells were harvested and washed 3 times in PBS and re-suspended in residual buffer. Cells were incubated with antibodies at dilution stated for 30 mins at +4oC and then washed in PBS and re-suspended in 100ul of PBS and stored on ice until analysis
Instrument	LSR Fortessa II, FACS-Aria-IIu, FACSAria-fusion (BD Biosciences)
Software	BD FACSDiva software
Cell population abundance	the studies were performed on homogenous cell populations for phenotypic analyses. The data reports specifically on the abundance of sub-populations within the NK cell population.
Gating strategy	Gates were established using unstained cells and FL-1 approaches

☒ Tick this box to confirm that a figure exemplifying the gating strategy is provided in the Supplementary Information.
